# Splenic diffuse red-pulp small B-cell lymphoma associated with hepatitis B virus: a report of two cases: Linfoma esplênico difuso da polpa vermelha, de linfócitos B pequenos, associado ao vírus da hepatite B: relato de dois casos

**DOI:** 10.1590/1516-3180.2016.0035130416

**Published:** 2016-07-18

**Authors:** Mariana Nassif Kerbauy, Carolina Melo Fernandes, Evandro Dantas Bezerra, Luis Alberto de Padua Covas Lage, Sheila Aparecida Coelho Siqueira, Juliana Pereira

**Affiliations:** I MD. Resident Physician, Department of Hematology and Hemotherapy, Faculdade de Medicina da Universidade de São Paulo (FMUSP), São Paulo, SP, Brazil.; II MD. Hematologist, Department of Hematology and Hemotherapy, Faculdade de Medicina da Universidade de São Paulo (FMUSP), São Paulo, SP, Brazil.; III MD, PhD. Professor in the Department of Pathology, Faculdade de Medicina da Universidade de São Paulo (FMUSP), São Paulo, SP, Brazil.; IV MD, PhD. Professor in the Department of Hematology and Hemotherapy, Faculdade de Medicina da Universidade de São Paulo (FMUSP - University of São Paulo), São Paulo, SP, Brazil.

**Keywords:** Lymphoma, Lymphoma, non-Hodgkin, Lymphoma, B-cell, Hepatitis B, Hepatitis B virus, Linfoma, Linfoma não Hodgkin, Linfoma de células B, Hepatite B, Vírus da hepatite B

## Abstract

**CONTEXT:**

Splenic diffuse red-pulp small B-cell lymphoma is a rare disease, representing less than 1% of all non-Hodgkin lymphomas (NHL). This entity is characterized by involvement of bone marrow sinusoids and peripheral blood. The majority of cases are at an advanced stage when diagnosed. Its pathogenesis is still poorly understood.

**CASE REPORTS:**

We report on two patients with chronic non-replicating hepatitis B virus (HBV) who developed splenic diffuse red-pulp small B-cell lymphoma. Both of them were in stage IV at diagnosis and evolved with aggressive disease. Both of them achieved a complete response through chemotherapy, but one of them died due to infectious complications during bone marrow transplantation. The other decided not to undergo transplantation and continues not to show any evidence of disease today (three years after treatment). Some studies have shown a possible association between B-cell NHL and HBV. Nonetheless, the mechanism through which this oncogenic virus interacts with B-cell NHL is still poorly understood. HBV is lymphotropic and may insert into the host’s genome, thus causing overexpression of oncogenes and downregulation of tumor suppressor genes. Therefore, chronic stimulation by HBV can increase B-cell proliferation, which promotes monoclonal expansion of these cells and results in malignancy.

**CONCLUSION:**

HBV may be implicated in the pathogenesis of this lymphoma, although no direct association between these two entities could be proved in the present study. Further investigations are necessary.

## RESUMO

**CONTEXTO:**

Linfoma esplênico difuso da polpa vermelha, de linfócitos B pequenos, é uma doença rara, representando menos do que 1% de todos os linfomas não Hodgkin. Essa entidade é caracterizada por envolvimento de sinusoides da medula óssea e sangue periférico. A maioria dos casos está em estádio avançado ao diagnóstico. Sua patogênese ainda é pouco compreendida.

**RELATOS DE CASOS:**

Reportamos dois pacientes com vírus da hepatite B (HBV) crônica não replicante que desenvolveram linfoma esplênico difuso da polpa vermelha, de linfócitos B pequenos. Ambos estavam em estádio IV ao diagnóstico e evoluíram com doença agressiva. Ambos alcançaram resposta completa com a quimioterapia, porém um deles evoluiu a óbito por intercorrências infecciosas durante o transplante de medula óssea e o outro optou por não realizar o transplante e encontra-se sem evidência de doença até os dias atuais (três anos após tratamento). Alguns estudos demonstraram a possível associação entre linfomas não Hodgkin B e HBV. Entretanto, o mecanismo pelo qual esse vírus oncogênico interage com linfoma não Hodgkin B ainda é pouco compreendido. HBV é linfotrópico e pode se inserir no genoma do receptor, causando superexpressão de oncogenes e *downregulation* de genes supressores tumorais. Portanto, o estímulo crônico pelo HBV pode aumentar a proliferação de células B, promovendo expansão monoclonal dessas células, resultando em malignidade.

**CONCLUSÃO:**

HBV pode estar implicado na patogênese desse linfoma, entretanto, uma associação direta entre essas duas entidades não pôde ser provada no presente estudo e investigações adicionais são necessárias.

## INTRODUCTION

Splenic diffuse red-pulp small B-cell lymphoma was recognized as a provisional entity in the 2008 update of the World Health Organization (WHO) classification.^1^ This new WHO update recognized two categories of primary splenic lymphomas; splenic marginal-zone lymphoma (SMZL) and the provisional group of splenic B-cell lymphoma/leukemia unclassifiable (SLLU), that includes splenic diffuse red-pulp small B-cell lymphoma and hairy-cell leukemia variant (HCL-v).^1^ The median age at which patients are diagnosed with splenic diffuse red-pulp small B-cell lymphoma is 72 years (range: 69-74 years),^2^ and the disease is seen predominantly among males, with a male/female ratio of 2.4:1.

Splenic diffuse red-pulp small B-cell lymphoma is a rare disease and represents less than 1% of all non-Hodgkin lymphomas (NHL) and 10% of B-cell lymphomas that are described in post-splenectomy series.^1^ This malignancy is characterized by diffuse infiltrate of monomorphic small to medium-sized B-cells with cytoplasmic villi, going into the red pulp of the spleen. The bone marrow sinusoids and peripheral blood are frequently involved. Patients usually present with an advanced stage of disease when they are diagnosed. Peripheral lymph node involvement and B-symptoms are rarely reported and massive splenomegaly is common.^1^ Splenic diffuse red-pulp small B-cell lymphomas have an indolent clinical course and there is no known standard treatment approach. So far, most of these patients have been treated with splenectomy.^3^

The pathogenesis of this lymphoma is still poorly understood and no risk factors associated with its development have been described. Recent studies have shown an association between B-cell NHL and hepatitis B virus (HBV), but the mechanism through which this oncogenic virus results in chronic lymphoproliferative B-cell disorders is not clearly understood. To the best of our knowledge, there is no case report in the literature correlating HBV with splenic diffuse red-pulp small B-cell lymphoma.

Here, we describe two cases of splenic diffuse red-pulp small B-cell lymphoma associated with chronic HBV infection.

## CASE REPORTS

### Case 1

A 62-year-old woman presented at our clinic complaining of weight loss, fever, night sweats and increasing abdominal size over the preceding two months. In her medical history, she reported having had rheumatoid arthritis, which was previously treated with chloroquine and methotrexate, and an untreated chronic non-replicating HBV infection (AntiHbC total +, AgHbe −, AntiHbe +, AgHbs +, AntiHbsAg -) with quantitative polimerase chain reaction (PCR) for HBV of 104 IU/ml (normal range of values: 20-170,000,000 IU/ml). Other viral serological tests for human immunodeficiency virus (HIV) and hepatitis C virus (HCV) were negative. In her family history, her daughter had had a central nervous system (CNS) neoplasm, a 60-year-old brother had had prostate cancer and another 50-year-old brother had died of acute leukemia.

**Figure 1.**
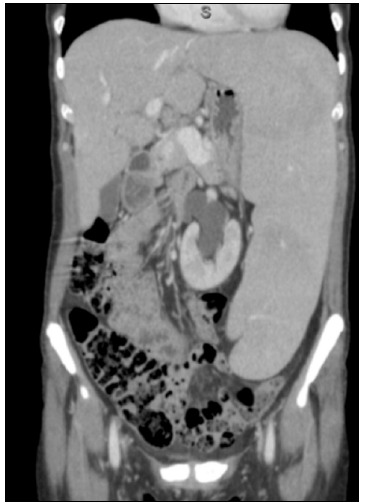
Computed tomography (CT) scan demonstrating subtle hepatomegaly and greatly increased spleen size.

In her physical examination, she presented as emaciated and pale, with massive splenomegaly and no palpable hepatomegaly or peripheral lymphadenopathy. Computed tomography (CT) scans showed subtle hepatomegaly and greatly increased spleen size, with no lymphadenopathy (Figure 1).

Laboratory tests showed normocytic and normochromic anemia (hemoglobin 8.8 g/dl, platelet count of 124 x 10^9^/l and total leukocyte count of 6.18 x 10^9^/l); 21% of the lymphoid cells had a high nuclear-to-cytoplasmic ratio, with loose chromatin, small clear nucleolus and thin cytoplasmic projections. Immunophenotyping of peripheral blood cells showed that they were positive for CD19, CD20, FMC7, CD22, and CD23, with strong expression of CD79b and CD25, weak surface expression of immunoglobulin (Ig) M, and partial expression of CD5. No expression of CD200, CD10, CD11c or CD103 was seen. Monoclonality was demonstrated through restriction of cytoplasmic light kappa chain Ig. Bone marrow biopsy revealed intrasinusoidal infiltration by small B lymphoid cells.

**Figure 2.**
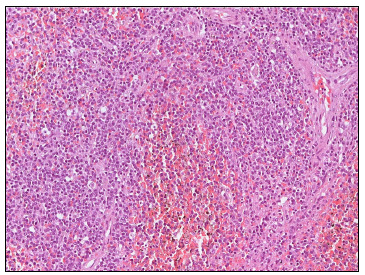
Diffuse infiltrate of small B-cell lymphocytes in the red pulp of the spleen (hematoxylin and eosin, x 200).

Splenectomy was performed as a therapeutic procedure and the histological analysis showed that the spleen weighed 1.818 kg, measured 27 x 14.3 x 8.3 cm and presented diffuse infiltrate in the red pulp of the spleen, with both cord and sinusoid infiltration by small B-cell lymphocytes (Figure 2) expressing CD20 and DBA-44 antigens. These cells had a low Ki67 proliferative index and were negative for CD3, CD5, CD10, CD23 and cyclin-D1. A hepatic biopsy revealed mixed inflammatory infiltration and stage 1 steatohepatitis, with an activity index of 5.

The patient improved clinically and her laboratory parameters normalized after splenectomy. However, 10 months later, the patient presented with weight loss, fever, night sweats, asymmetrical eyelid edema and pleural effusion. At that time, laboratory examinations showed leukocytosis of 30.18 x 10^9^/l; 22% of the cells were of moderate size and presented loose chromatin and evident peripheral nucleoli, which were morphologically suggestive of centroblasts. Her laboratory values showed hypercalcemia and elevated lactate dehydrogenase (LDH), of more than 3,000 U/l (normal values range from 240 to 480 U/l). Pleural fluid showed infiltration by neoplastic cells. New immunophenotyping of peripheral blood cells revealed aberrant lymphoid cells expressing B antigens, including CD19, surface IgM (sIgM), strong CD20 expression, heterogeneous partial CD79b expression and FMC7 with a lack of CD5 antigen. Bone marrow karyotyping revealed 46,XX [20]. The bone marrow biopsy was hypercellular due to infiltration by B cells.

Brain and orbit magnetic resonance imaging (MRI) showed infiltration of the optic nerve, bilateral lacrimal glands and CNS. Restaging CT scans revealed renal infiltration and axillary and abdominal lymph node enlargement. It was concluded that transformation to high-grade B-cell lymphoma had occurred, and treatment with R-CHOP was started, with the following given on day 1 of each cycle: intravenous rituximab, 375 mg/m^2^; intravenous cyclophosphamide, 750 mg/m^2^; intravenous doxorubicin, 50 mg/m^2^; and intravenous vincristine, 1.4 mg/m^2^. In addition, oral prednisone, 100 mg once daily, was given on days 1-5. R-CHOP was administered in combination with intrathecal chemotherapy containing methotrexate (12 mg), cytarabine (60 mg) and dexamethasone (2 mg) (MADIT) and two cycles of methotrexate (3 g/m^2^ intravenously) after the last cycle of R-CHOP and radiotherapy to the orbits (24 Gy). Tenofovir (300 mg/day) was started along with the chemotherapy to avoid replication of HBV. The patient achieved a complete response, and autologous bone marrow transplantation was performed as a consolidation strategy. However, unfortunately, the patient died due to infectious complications during the transplantation.

### Case 2

A 29-year-old female patient with a seven-year history of splenomegaly, without portal hypertension and with chronic non-replicating HBV infection (AntiHbC total +, AntiHbe +, AgHbe −, AgHbs +, AntiHbs −) showing quantitative PCR for HBV of less than 20 IU/ml (normal range of values: 20-170,000,000 IU/ml) presented to our service complaining of progressive spleen enlargement and B symptoms (fever, night sweats and weight loss) for the past two months. Laboratory tests showed hemoglobin 8.1 g/dl, platelets 80 ×10^9^/l and white blood cells 12 × 10^9^/l, with 85% atypical lymphoid cells. Immunophenotyping demonstrated strong expression of CD45, positivity for the B-cell antigens CD20 and CD19, weak expression of CD25, partial expression of CD23 and CD200 and negativity for CD11c and CD103. Monoclonality for kappa light chain Ig was demonstrated in both the peripheral blood and bone marrow.

**Figure 3.**
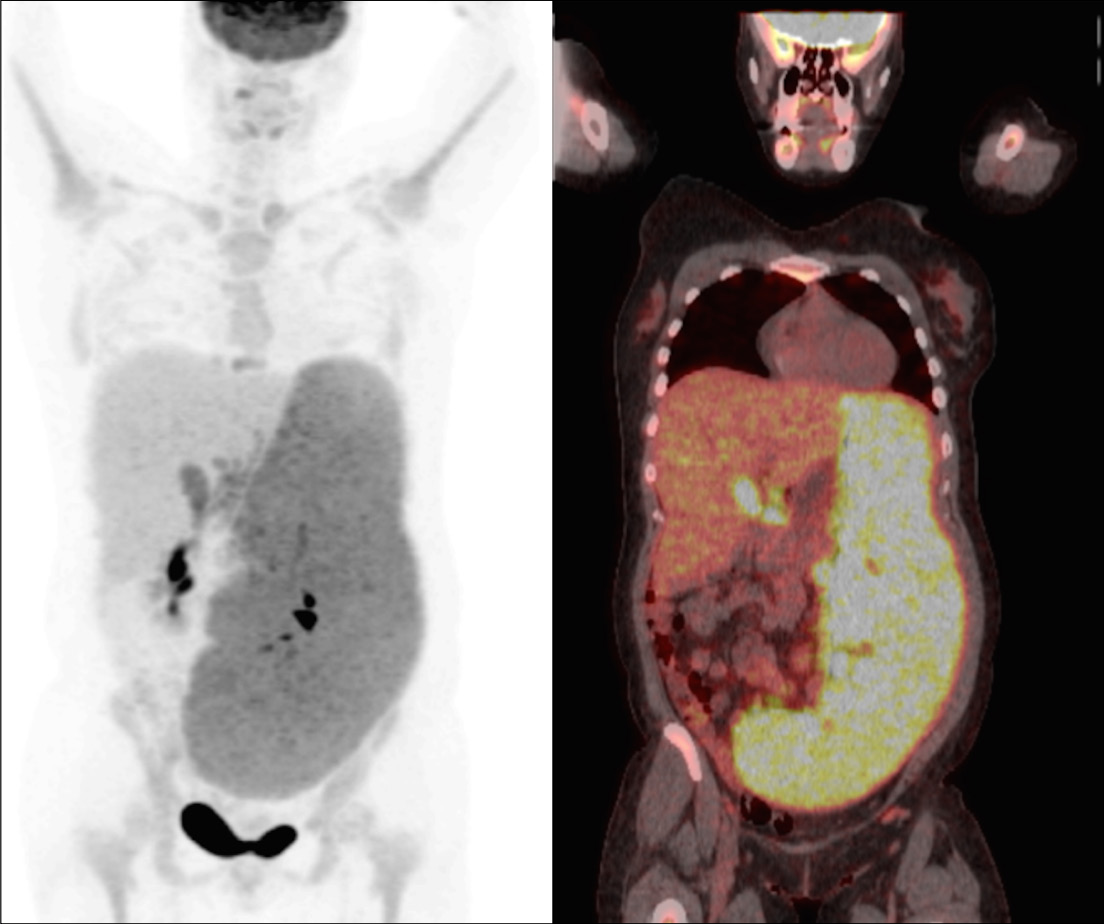
18FDG-PET-CT (fludeoxyglucose F18 position emission computed tomography) demonstrating major splenomegaly (standard uptake value [SUV]: 6.1) and hepatomegaly (SUV: 3.2)

The karyotype was complex, with structural abnormalities in the 14q32 region, deletions on chromosomes 6 and 7, isochromosomes 8 and 17 and trisomy 18. Fluorescence in situ hybridization analysis revealed an extra copy of the MYC gene and a p53 deletion. ^18^FDG-PET-CT showed splenomegaly (standard uptake value [SUV]: 6.1), hepatomegaly (SUV: 3.2), abdominal lymph node enlargement (SUV: 6.3) and cervical and mediastinal lymphadenopathy (Figure 3).

**Figure 4.**
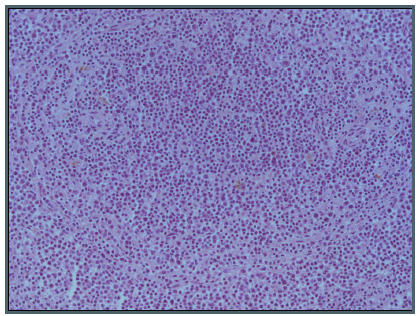
Small and few large lymphoid cells in splenic red pulp (hematoxylin and eosin, x 200)

Splenectomy was indicated and the anatomopathological analysis showed that the spleen dimensions were 33.0 x 20.6 x 7.5 cm. The spleen showed infiltration by small to intermediate-sized mature lymphoid cells, with large cells among them, primarily in the red pulp and sinusoidal space of the spleen and secondarily in the white pulp of the spleen (Figure 4). Immunohistochemical analysis showed expression of CD20 and DBA44, with Ki67 of 40%. The cells were negative for CD5, CD10, CD23, CD3 and cyclin D1. A hepatic biopsy demonstrated nodular lymphoid infiltrate involvement, with suspected lymphoid neoplasia. A hilum splenic lymph node (measuring 2.6 x 1.1 x 1.0 cm) showed lymphoid neoplasia of mature cells with predominance of intermediate to large cells with a nodular pattern.

The diagnosis of splenic diffuse red-pulp small B-cell lymphoma was made and treatment was then instituted, consisting of eight cycles of R-CHOEP: intravenous rituximab (375 mg/m^2^), intravenous cyclophosphamide (750 mg/m^2^), intravenous doxorubicin (50 mg/m^2^), and intravenous vincristine (1.4 mg/m^2^) on day 1; intravenous etoposide (100 mg/m^2^) on days 1-3; and oral prednisone (100 mg orally once daily) on days 1-5. It was proposed that this would be followed by autologous stem cell transplantation. After eight cycles of R-CHOEP, the patient achieved complete response, but she became pregnant and chose not to undergo the transplantation. Nonetheless, she continues not to present any evidence of disease today (three years after the treatment). Administration of tenofovir (300 mg/day) was started along with the chemotherapy.

## DISCUSSION

We have reported on two patients with chronic non-replicating HBV who developed splenic diffuse red-pulp small B-cell lymphoma. The diagnosis of primary chronic lymphoproliferative disorders of the spleen is established based on analysis on splenectomy tissue, or on integration of clinical data, assessment of lymphocyte morphology in peripheral blood, immunophenotyping of these lymphocytes and evaluation of bone marrow infiltration pattern from biopsy. For our patients, we used both criteria in order to be sure of the diagnosis. In both cases, the splenectomy product demonstrated diffuse neoplastic infiltration consisting of small mature lymphocytes with primary immunophenotype B of the red pulp of the spleen. With this presentation, the diagnosis of splenic marginal-zone lymphoma was ruled out, since this entity is characteristically a primary neoplasm of the splenic white pulp, usually with nodular architectural arrangement.

Other entities that might have formed differential diagnoses were hairy-cell leukemia and hairy-cell leukemia variant. However, the clinical data in association with morphological and immunophenotyping data made it possible to safely rule out diagnoses of hairy-cell leukemia (absence of pancytopenia, no cells with the classical morphology of “hairy cell” and immunophenotyping not characterized by strong expression of CD11c, CD25, CD103 and CD123 antigens) or hairy-cell leukemia variant (no lymphocytes with morphology characterized by the presence of central vesicular nucleolus, cytoplasm similar to hairy cell and absence of strong expression of CD11c and CD20 antigens).

Thus, integration of all these findings, i.e. lymphoproliferative malignancy of small B cells, diffuse infiltration of the spleen and primary from splenic red pulp, with exclusion criteria for “hairy cell” and its variant form, enabled the definitive accurate diagnosis of splenic diffuse red-pulp small B-cell lymphoma in both cases reported here.

We conducted a systematic search in the main electronic databases (PubMed, Google Scholar and Lilacs Library), to find articles relating to splenic diffuse red-pulp small B-cell lymphoma in association with hepatitis B infection. In order to make the search as wide as possible, no limits were applied regarding the date of publication, the language or the research design (Table 1). There were fewer than 100 search results and none of them described any association between splenic diffuse red-pulp small B-cell lymphoma and hepatitis B.

**Table 1.**
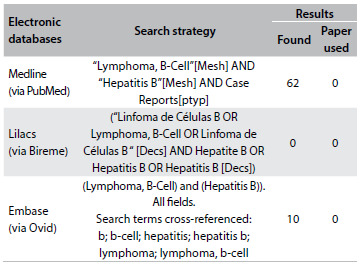
Systematic search of the literature performed on April 4, 2016

Mollejo et al. first proposed this lymphoma as a new subtype in 2002,^4^ consequent to reviewing 85 cases of SMZL. These authors identified four cases with predominance of monomorphic infiltration in the red pulp of the spleen instead of the typical micronodular component in the white pulp commonly seen in SMZL. Later on, two series of cases of splenic diffuse red-pulp small B-cell lymphoma were published by Traverse-Glehen et al.^5^ and Kanellis et al.^2^

Similarly to our two patients, all cases previously reported were also in stage IV at diagnosis. Diffuse infiltration of the red pulp of the spleen, affecting both cords and sinuses, along with marked DBA-44 positivity, was frequently found by Kanellis et al.^2^ (88.2% of their cases), as well as in our cases. Additionally, previous studies described situations of lymph node replacement by diffuse neoplastic infiltrate, with preserved sinuses.^2^^,^^4^

The most common finding in the bone marrow biopsies was predominance of intrasinusoidal lymphoid infiltration, sometimes associated with interstitial and nodular involvement, along with hematopoietic tissue and absent or mild fibrosis. The malignant cells presented with round to slightly irregular nuclei, vesicular chromatin and moderate amounts of pale cytoplasm and cytoplasmic projections similar to SMZL villous cells. These cells expressed CD20, DBA44 and IgG. Presence of annexin A1, CD25, CD3, CD5, CD23, CD103, CD123, CD11c, CD38, CD10, Bcl6, bcl2, cyclin D1 and IgD was uncommon.^1^^,^^4^ Our cases are consistent with those presented in the literature regarding the pathological and immunophenotypic findings of splenic diffuse red-pulp small B-cell lymphoma. The majority of the cases reported in the literature had a normal karyotype and the most frequent chromosomal abnormalities were 7q and 3q deletions and trisomy 18.^1^^,^^6^ One of our cases showed a complex karyotype.

Kanellis et al.^2^ found p53 inactivation in all cases of splenic diffuse red-pulp small B-cell lymphoma, comprising either p53 mutation (2/4 cases) or anomalous p53 staining. Mollejo et al.^4^ reported that TP53 abnormality was present in 2 out of 13 cases (15.3%): one of these patients showed disease progression and ultimately died of the disease. In the same way as described by these authors, one of our patients also had a p53 deletion and evolved to aggressive disease. Splenic diffuse red-pulp small B-cell lymphoma is an indolent lymphoma and patients can be maintained using a watchful waiting approach or may undergo splenectomy.

Compared with SMZL, splenic diffuse red-pulp small B-cell lymphoma has demonstrated better disease-free survival, but overall survival is not statistically different. At diagnosis, our patients presented with anemia, which is associated with worse prognosis according to Kanellis et al.,^2^ and both of them had HBV infection as a comorbidity. HBV infection may be associated with worse prognosis, since both patients evolved with aggressive disease.

Virus-induced carcinogenesis is known to occur in several lymphoid malignancies in which the virus has a pathogenic role, such as in Burkitt’s lymphoma with Epstein-Barr virus (EBV), adult T-cell leukemia with human T-cell lymphotropic virus-1, and primary effusion B-cell lymphoma with human herpes virus 8. Some malignancies may be caused by chronic stimulation such as in mucosa-associated lymphoid tissue lymphoma, which is associated with *Helicobacter pylori*, or through an indirect mechanism. While EBV has a direct carcinogenic effect on Burkitt’s lymphoma through activating the c-Myc oncoprotein, HIV acts on the immune system to reduce immune surveillance.^7^^,^^8^

HBV is a small DNA virus that is a member of the Hepadnaviridae family. It replicates through a RNA intermediary and can integrate into the host genome. HBV infection is highly prevalent worldwide, with around 350 million chronically infected individuals. It is endemic in Asia, Africa, the Middle East, Eastern Europe and South America.^9^ There are 500,000 to 1.2 million deaths due to chronic hepatitis B annually, out of a total of 350 million cases worldwide,^8^ and about 340,000 cases of liver cancer relating to HBV.^10^

HBV and HCV are known to induce acute and chronic hepatitis and are strongly associated with hepatocellular carcinoma (HCC). HBV carriers have a 200 times higher risk of HCC, and this is one of the highest risks for a human malignancy.^11^ Its pathogenesis involves multiple pathways, including oxidative stress, hepatic inflammation leading to genetic damage and integration of HBV DNA into the host genome, thereby leading to genetic alterations such as chromosomal and gene translocations and deletions or generation of fusion transcripts. These alterations may change oncogenes, tumor-suppressor genes and expression of small non-coding RNA molecules (miRNAs).^12^

Non-structural HBV X protein is a key regulatory protein that modulates viral replication and pathogenesis. It is transcribed in human HCC tumor cells, even when HBV replication is absent, due to viral DNA integration. This protein modulates gene expression, since it interacts with transcription factors, activates mitogenic signaling cascades and interacts with cellular proteins, including p53. Consequently, DNA repair, tumor suppressor genes and the cell cycle could become deregulated.^13^

The relationship between NHL and HCV has already been proven, with evidence demonstrating a causal relationship.^11^^,^^14^^,^^15^^,^^16^^,^^17^ There is a known multicausal process involving direct lymphocytic activation mediated by viral proteins and chronic antigenic stimulation, for promoting B-cell transformation.^18^ A recent prospective cohort study conducted in South Korea evaluated 603,585 participants, among whom 53,045 (9%) tested positive for HBsAg. The HBsAg-positive patients were at higher risk of NHL than those who were HBsAg-negative (hazard ratio: 1.74; 95% CI: 1.45-2.09), especially for diffuse large B-cell lymphoma and immunoproliferative diseases. In this study, HBsAg was not associated with follicular lymphoma, T-cell NHL, Hodgkin’s lymphoma, multiple myeloma or leukemia.^19^ A meta-analysis on 17 case-control and five cohort studies found that HBV-infected individuals had an odds ratio (OR) of 2.24 (95% CI: 1.80-2.78; P ≤ 0.001) for developing NHL.^20^ Another meta-analysis on 12 case-control studies, comprising 11 studies evaluating HBV infection in NHL and one study that had investigated NHL in HBV infection, reported that the OR of detecting HBV infection in lymphoma patients was 2.5 times higher than in controls.^21^ These studies suggest a possible relationship between HBV and NHL. The pathogenic association is less well studied than relationships with HCV, but it appears to result from different HBV-driven events.^22^

HBV is a hepatotropic virus and replicates within hepatocytes, but it can also cause lymphotropism for lymphocytes in the peripheral blood, bone marrow, spleen, lymph nodes, and thymus.^23^^,^^24^^,^^25^^,^^26^ This lymphotropism of HBV is a fundamental property favoring causality between HBV and B-cell malignancies. Additionally, a study evaluating CD20 cell expression in the liver of HBsAg-positive and HBeAg-negative patients found that all of them were CD20-positive.^27^

Wang et al.^28^ found higher prevalence of HBeAg and anti-HBe in NHL cases than in controls, thus suggesting that viral replication may be required to support neoplastic proliferation. HBV integrates into the host genome and can lead to overexpression of cellular oncogenes or downregulation of tumor suppressor gene expression. Introduction of whole-genome sequencing identified several preferred sites for HBV integration, some of which have also been described in lymphomas, CCNE (cyclin E1)^29^ and the PDGF receptor.^30^ Also, the HBX protein transactivates cellular promoters and enhancers, including the binding site for NF-KB.^12^ The HBX protein is involved in blocking p53-mediated apoptosis, which has been implicated in the pathogenesis of splenic diffuse red-pulp small B-cell lymphoma.^12^^,^^28^^,^^31^^,^^32^ A second mechanism for lymphomagenesis could relate to chronic stimulation by HBV antigens, thereby leading to incremental proliferation of B-lymphocytes and, consequently, monoclonal malignant expansion. The incremental B-cell proliferation may lead to predisposition towards genetic aberrations, thus promoting neoplastic transformation.^28^

## CONCLUSION

We have described two cases of splenic diffuse red-pulp small B-cell lymphoma associated with HBV. Although we hypothesized that HBV might be implicated in the pathogenesis of this lymphoma, no direct association between these two entities could be proved in this study, and further investigations are necessary.
